# The Yellow Fever

**Published:** 1898-03

**Authors:** 


THE YELLOW FEVER.—A feeling of insecurity as to the recurrence of yellow fever the coming summer is manifest among the timid. The fact that the past winter was so mild, there not being a real good freeze to kill the germs, causes many to fear that, hibernating in New Orleans, it will break out early this summer. To that end the Florida health authorities will put on general quarantine April 1st, instead of May 1st, the usual time when all States declare quarantine. Alabama also contemplates a similar action. To do this, pending the investigation into the destruction of the Maine, will give rise to much embarrassment, and the naval officers are already kicking against it. We are not prepared to say whether or not Texas will take a similar step.

---

COCK'S ANTI-BACILLA COMPOUND, the new remedy for consumption, to which we called attention last April, will shortly be offered to the medical profession through the big drug firm of I. L. Lyons, New Orleans, who, we learn, have entered into a contract with Dr. Cock to manufacture it and put it on the market. It will be offered only through the medical profession. It is claimed for this remedy the most brilliant results at the hands of well known Texas practitioners. Dr. Cock will make New Orleans his future home.

---

THE JOURNAL is pained to announce the serious illness of our universally esteemed State Health Officer, Dr. Swearingen. At this writing he is improving and his friends have hopes of his early recovery.

